# Assessment of vascular reactivity in rat brain glioma by measuring regional blood volume during graded hypoxic hypoxia

**DOI:** 10.1038/sj.bjc.6601908

**Published:** 2004-06-08

**Authors:** C Julien, J-F Payen, I Troprès, R Farion, E Grillon, O Montigon, C Rémy

**Affiliations:** 1 Unité Mixte INSERM/UJF 594, LRC CEA 30 V, Pavillon B, BP 217, Hôpital Albert Michallon, F-38043 Grenoble, France; 2 Département d'Anesthésie-Réanimation, Hôpital Albert Michallon, F-38043 Grenoble, France

**Keywords:** cerebral blood volume, brain, magnetic resonance imaging, tumour, glioma, hypoxia

## Abstract

While morphological and molecular events during angiogenesis in brain glioma have been extensively studied, the functional properties of tumour vessels have yet received little attention. We have determined changes in regional blood volume (BV) during graded hypoxic hypoxia using susceptibility contrast magnetic resonance imaging in a model of rat brain glioma. Nine anaesthetised and ventilated rats with C6 glioma were subjected to incremental reduction in the fraction of inspired oxygen (FiO_2_): 0.35, 0.25, 0.15, 0.12, 0.10 and reoxygenation to 0.35. At each episode, BV was determined in peritumoral, intratumoral and contralateral regions. Baseline BV values (FiO_2_ of 0.35) were higher in peritumoral than in the contralateral and intratumoral regions. Progressive hypoxia resulted in a graded increase in BV in contralateral and peritumoral regions. At FiO_2_ of 0.10, BV increases were comparable between these two regions: 49±22% (s.d.) and 28±17% with respect of control values, respectively. These BV changes reversed during the reoxygenation episode. By contrast, the intratumoral region had a significant increase in BV at FiO_2_ of 0.10 only, with no evidence of return to the basal value during reoxygenation. Immunohistochemical staining of *α*-smooth muscle actin confirmed reactivity of vessels in the peritumoral region. Our findings indicate that peritumoral vessels present a vascular reactivity to hypoxia, which is comparable to that of nontumoral vessels. A method is thus available for noninvasively demonstrating whether any particular vascular modifying strategy results in the desired outcome in terms of tumour blood volume changes.

Mechanisms of brain glioma proliferation have been extensively studied. This tumour grows initially by cooptation of existing host vessels (Holash *et al*, 1999). In a second phase, the host vessels regress, leading to a secondary avascular tumour and eventually to angiogenesis. The switch to an angiogenic phenotype results from a positive balance between proangiogenic factors, for example, vascular endothelium growth factor (VEGF) and antiangiogenic factors (Folkman and Klagsbrun, 1987). Hypoxia may play a major role in the induction of the angiogenic switch (Shweiki *et al*, 1992), as hypoxic perinecrotic regions and VEGF expression have been closely related (Damert *et al*, 1997; Rofstad and Danielsen, 1999). Vascular endothelium growth factor produced in the hypoxic region induces formation of neovessels in the peritumoral region. Three regions can be thus distinguished during advanced development of brain glioma: a central region with necrosis and no vessels; a peritumoral region with a high degree of angiogenesis, including tumour tissue and nontumour tissue at the edge of the tumour and containing tumour cells; and an intermediate region with large, tortuous and pleomorphic vessels (Deane and Lantos, 1981; Zama *et al*, 1991).

Few studies have been aimed at investigating the functional properties of the vessels in brain glioma (Mazurchuk *et al*, 1999). Yet, the possibility to enhance local blood flow in the tumoral tissue presents interest in view of enhancing drug delivery to the tumoral tissue. One way to explore the cerebrovascular reactivity is to measure blood volume (BV) response to hypoxic hypoxia (low PaO2), which is a potent vasodilator in brain (Kontos *et al*, 1978). We recently measured regional cerebral BV response to hypoxic hypoxia in normal rats, using susceptibility contrast magnetic resonance imaging (MRI) (Julien-Dolbec *et al*, 2002). By measuring BV response to hypoxic hypoxia in a model of rat brain glioma, our goal was to assess overall vessel reactivity to hypoxia in those tumours and to determine possible differences in responsiveness between intratumoral, peritumoral and nontumoral areas. We defined peritumoral region as the region at the edge of the tumour having the largest BV due to a high vessel density.

## MATERIALS AND METHODS

### Animal preparation

Animals were prepared in accordance with the guidelines of the French Government (decree No. 87–848 of 19 October 1987, licenses 006683 and A38071) and with the UKCCCR Guidelines for the Welfare of Animals in Experimental Neoplasia (Workman *et al*, 1998). A total of 26 Wistar female rats (160–180 *g*) were studied. For the purpose of tumour implantation, rats were anaesthetised with an intraperitoneal injection of chloral hydrate (400 mg kg^−1^). A cell suspension of C6 glioma (10^5^ cells in 5 *μ*l of a culture medium without foetal calf serum) was stereotaxically injected using a Hamilton syringe through a burr hole (1 mm diameter) in the right striatum (7 mm anterior to the zero ear bars, 3 mm right to the midline, 3 mm depth from the dura). The C6 glioma cell line was established by Benda *et al* (1971) from a methyl-nitrosourea-induced rat glioma. The C6 cells were cultured in Dulbeco's modified Eagle's medium (DMEM, GilboBRL, Lifes technologies, Scotland) supplemented with 10% foetal calf serum (GilboBRL, Lifes technologies, Scotland), 50 U ml^−1^ penicillin and 50 *μ*g ml^−1^ streptomycin.

The MRI study was performed during the 4th week after tumour implantation, so that significant tumour growth and brain neovessels could be observed (Peoc'h *et al*, 1999). Anaesthesia was induced with 4% halothane and then maintained with an intraperitoneal injection of thiopental (20 mg kg^−1^). In total, 1% lidocaine was injected subcutaneously for local anaesthesia at all surgical sites. After tracheostomy, rats were mechanically ventilated with 65% nitrous oxide–35% oxygen using a rodent ventilator (Model 683, Harvard Apparatus Inc., South Natick, MA, USA). Ventilation was adjusted to maintain partial arterial CO_2_ pressure (PaCO_2_) at ≈35 mmHg. The fraction of inspired oxygen (FiO_2_) was continuously monitored (MiniOX I analyser, Catalyst Research Corporation, Owings Mills, MD, USA). A 0.7-mm indwelling catheter was inserted into the left femoral artery to monitor mean arterial blood pressure (MABP) via a chart recorder (8000S, Gould Electronic, Ballainvilliers, France). Blood gases (PaO_2_ and PaCO_2_), arterial saturation of haemoglobin in oxygen (SaO_2_), arterial pH (pHa) and haemoglobin content (Hb) were analysed from less than 0.1 ml arterial blood samples (ABL 510, Radiometer, Copenhagen, Denmark). Another 0.7-mm indwelling catheter was inserted into the left femoral vein to continuously infuse a normal saline solution containing epinephrine (25 ng min^−1^) and sodium bicarbonate (0.4 *μ*mol min^−1^) at a rate of 2 ml h^−1^ throughout the study. Epinephrine was required to protect from the adverse effects of combined anaesthesia and hypoxic hypoxia on the cardiovascular system. Sodium bicarbonate was used to prevent arterial acidosis. Canulation of the femoral vein was also required for the injection of the contrast agent. Rectal temperature was maintained at 37.5±0.5°C by using a heating pad placed under the abdomen.

### Experimental protocol

**Figure 1:**
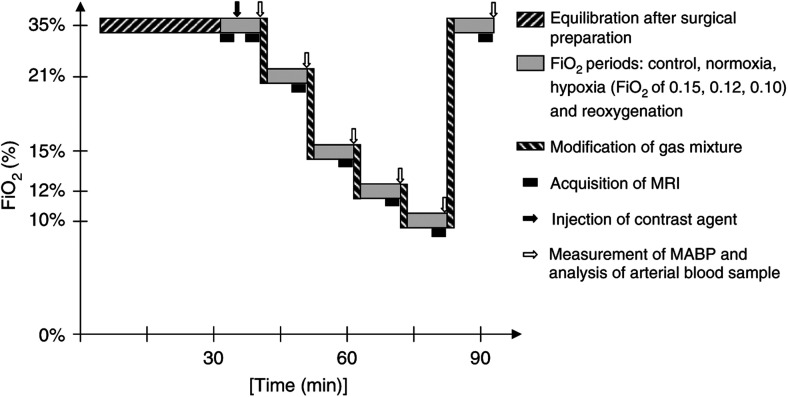
Experimental protocol for the BV measurements in rat brain tumour during graded hypoxic hypoxia. Magnetic resonance imaging was performed before injection of contrast agent at the beginning of the control episode. Postcontrast MRI, mean arterial blood pressure (MABP) measurement and arterial blood gas analysis were performed 4 min after onset of each FiO_2_ level.

Animals were subjected to a stepwise lowered FiO_2_: control episode (FiO_2_ of 0.35), normoxia episode (FiO_2_ of 0.25), hypoxic episodes (FiO_2_ of 0.15, 0.12 and 0.10) and reoxygenation episode (FiO_2_ of 0.35) (Figure 1

). The basic cycle started after a 30 min equilibration episode at FiO_2_ of 0.35 (control). The initial criteria for exclusion from the study were: MABP <100 mmHg, pHa <7.30, PaO_2_ <100 mmHg, Hb <10 g dl^−1^. The subsequent episodes were then first induced by lowering the inhaled oxygen for FiO_2_=0.25, then by replacing the oxygen by air (FiO2 of 0.15 and 0.12). During these four episodes, fractions of inspired nitrous oxide were 0.65, 0.75, 0.25 and 0.40, respectively. Reoxygenation was obtained by restoring the oxygen in the gas mixture. Each FiO_2_ episode (normoxia, hypoxia and reoxygenation) lasted 10 min: a 4 min equilibrium period followed by MRI acquisition and determination of MABP and arterial blood sampling. If MABP <70 mmHg, pHa <7.30 or PaCO_2_ <27 mmHg at FiO_2_ of 0.25, 0.15 and 0.12, the animal was excluded from the study. No exclusion criterion was applied at FiO_2_ of 0.10 (severe hypoxia) and during the reoxygenation episode. When the cycle of measurements ended, rats were killed by administration of an overdose of thiopental (50 mg kg^−1^). Brains were excised from the skull for histological study, and frozen in isopentane at −50°C and stored at −80°C.

### Magnetic resonance imaging measurement

Magnetic resonance imaging was performed with a 2.35 T, 40-cm diameter horizontal bore magnet (Bruker Spectrospin, Wissenbourg, France) equipped with a 20-cm diameter actively shielded gradient (Magnex Scientific Ltd, Adingdon, UK) and with a SMIS console (SMIS Ltd, Guildford, UK). The rat was lying prone, its head secured via ear bars. A 30-mm diameter surface coil was located directly above the brain. After radiofrequency coil matching and tuning, the magnetic field homogeneity was adjusted to obtain a linewidth for water smaller than 0.5 part per million in the brain. Six adjacent horizontal slices (from 2 mm below bregma) were chosen from a coronal T_1_-weighted image. A series of T_2_^*^-weighted images for each slice was acquired at different echo times using a multigradient-echo sequence with an echo spacing interval of 4.2 ms (repetition time *T*_R_=2,000 ms; first echo time *T*_E_=7.6 ms; 64 × 32 data acquisition image matrix; number of averages=2; field of view=35 × 35 mm^2^; slice thickness=1 mm; number of slices=6). Acquisition of all images of the six slices took about 3 min.

A superparamagnetic iron oxide contrast agent was intravenously injected (200 *μ*mol of iron per kg body mass of AMI-227, Sinerem®; Guerbet, Aulnay-sous-Bois, France) 30 min after the start of the experiment (FiO_2_ of 0.35). Images were acquired before (*n*=24 echoes, precontrast image) and 3 min after injection (*n*=12 echoes, postcontrast image). Acquisition of postcontrast images was then repeated at the end of each subsequent FiO_2_ episode (FiO_2_ of 0.25, 0.15, 0.12 and 0.10, and reoxygenation).

### Magnetic resonance imaging analysis

Image processing and determination of BV were performed using an Ultrasparc workstation (Sun Microsystems, Pasadena, CA, USA). Susceptibility contrast MRI exploits the increase in the magnetic susceptibility difference (Δ*χ*) between the intravascular and the extravascular compartments induced by the presence of a long-lived intravascular contrast agent. This increase in Δ*χ* results in an increase Δ*R*_2_^*^ of the transverse decay rate (*R*_2_^*^=1/*T*_2_^*^) of the NMR signal from the water protons. Δ*R*_2_^*^ is proportional to BV as previously shown (Yablonskiy and Haacke, 1994; Boxerman *et al*, 1995; Tropres *et al*, 2001):


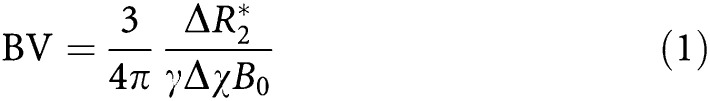


where *γ* is the gyromagnetic ratio, Δ*χ* the susceptibility difference, *B*_0_ the magnetic field in the absence of sample. For an injection of AMI-227 of 200 *μ*mol of iron per kg, Δ*χ* was equal to 0.688 part per million at 2.35 T in large vessels (Tropres *et al*, 2001). Since measurements were performed in brain microcirculation, Δ*χ* value was corrected by the ratio of haematocrit between brain microcirculation and large vessels that corresponded to 0.83 (Bereczki *et al*, 1993). This resulted in a Δ*χ* value of 0.571 (Payen *et al*, 1998). We also assumed that the brain haematocrit remains constant during hypoxic hypoxia, as previously shown in most brain areas (Bereczki *et al*, 1993). Regional BV is expressed as the fractional blood volume in each voxel, or in ml 100 g^−1^ tissue.

**Figure 2:**
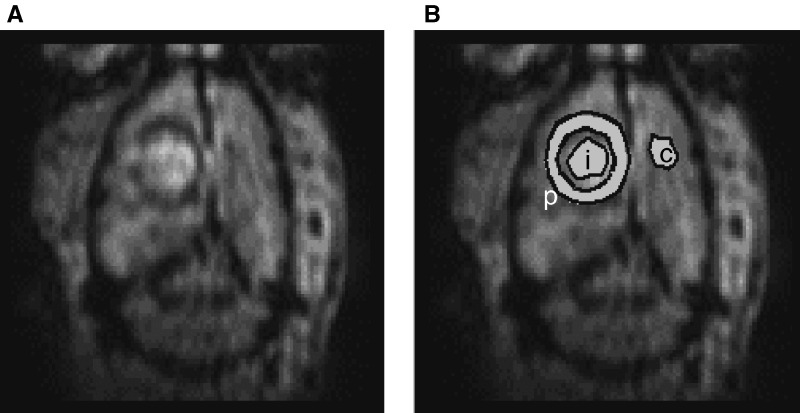
Regions of interest (ROI) defined on *T*_2_^*^-weighted images: peritumoral (p), intratumoral (i) and contralateral (c) regions. The *T*_2_^*^-weighted images (first echo) acquired at FiO_2_ of 0.10 (**A**) were used to select ROI (**B**).

**Figure 3:**
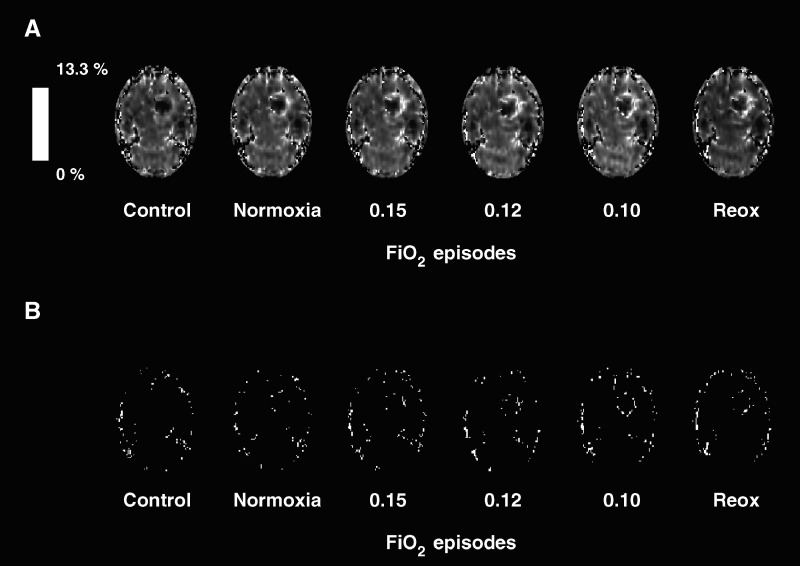
(**A**) Example of Δ*R*_2_^*^ maps for control episode (FiO_2_ of 0.35), normoxia episode (FiO_2_ of 0.25), hypoxia episodes (FiO_2_ of 0.15, 0.12, 0.10) and reoxygenation episode (Reox, FiO_2_ of 0.35). (**B**) Pixels discarded from BV measurement because of excessive Δ*R*_2_^*^ values.

For each FiO_2_ episode, *T*_2_^*^ maps were calculated by a least-squares monoexponential fit of the signal intensity *vs* the echo time on a pixel by pixel basis. Differences in relaxation rates in each pixel were then calculated according to the formula:


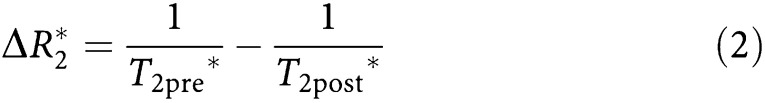


with *T*_2 pre_^*^ and *T*_2 post_^*^ are the relaxation times before and after administration of the contrast agent, respectively. The Δ*R*_2_^*^ values were measured during the six successive FiO_2_ episodes. Three regions of interest (ROI) were defined on the *T*_2_^*^-weighted images (first echo) acquired at FiO_2_ of 0.10 (Figure 2

): peritumoral region (≈192 pixels) corresponding to the hyposignal at the edge of the tumour on the *T*_2_^*^-weighted images, intratumoral region (≈62 pixels) corresponding to hypersignal inside the tumour on the *T*_2_^*^-weighted images and contralateral (striatum) region (≈44 pixels). Pixels with excessive Δ*R*_2_^*^ values (>200 s^−1^), that is, BV larger than 13.3 ml.100g^−1^, during any FiO_2_ episode were discarded (Figure 3B

). This threshold corresponds to the upper limit of validity of the linear relationship between Δ*R*_2_^*^ and BV. Selection of the ROI was made at FiO_2_ of 0.10 because it corresponded to the highest number of pixels to be discarded. A correction for clearance of the contrast agent from the plasma (elimination half-time ≈4.5 h) was applied since the postcontrast experiments lasted ≈75 min. This correction has been described elsewhere (Payen *et al*, 1998).

### Histological study

Two groups of excised brains were sliced in horizontal plane using a cryotome (10 *μ*m thick). The first group included all rat brains studied by MRI. A total of 24 brain sections located in the MRI slice were obtained every 250 *μ*m. Haematoxylin erythrosin safran (HES) staining was used to characterise the brain tumour (localisation, size and regions). In the second group of excised brains (rats excluded from the MRI study due to the physiological inclusion criteria), three adjacent brain sections were obtained from the largest tumour surface. One of these sections was stained using HES to characterise brain tumour. Another brain section was immunostained using goat anti-collagen primary antibody (1 : 100; 1 h at 4°C) (Southern Biotechnology Associates, Birmingham, AL, USA) and biotinylated anti-goat secondary antibody (1 : 500; 30 min at room temperature) (Santa Cruz Biotechnology, Santa Cruz, CA, USA) to identify mature and immature brain vessels. The last brain section was immunostained using mouse anti-*α* smooth muscle actin (*α*-SMA) primary antibody (1 : 500; 1 h at 4°C) (Dako, Glostrup, Denmark) and biotinylated anti-mouse secondary antibody (1 : 1000; 30 min at room temperature) (Valbiotech, Paris, France) to identify immature brain vessels. Biotinylated antibodies were detected using extravidin-peroxidase (1 : 1000; 30 min at room temperature) (Sigma-Aldrich, Saint-Quentin Fallavier, France). The sections were rinsed three times with phosphate-buffered saline (Sigma-Aldrich, Saint-Quentin Fallavier, France) between incubations. The sections were analysed using an optical microscope at low and high magnification (× 100 and × 200). Histological analysis was qualitative and allowed verification of brain tumour growth, confirmation of the ROI selection made for MRI analysis, and documentation of tumour vascular architecture.

### Statistical analysis

Data are expressed as mean±s.d. Analysis for statistical significance of changes during the successive episodes was performed using one-way analysis of variance (ANOVA) for repeated measurements (StatView SE program, Abacus Concepts Inc., California, USA). To examine regional differences in the response to hypoxia, interaction between brain regions and episodes was assessed using two-way ANOVA (ROI × FiO_2_ episodes) for repeated measurements. Each value at a given episode was compared to that obtained at another episode using the Scheffé *post hoc* test. Difference in BV between brain regions at each FiO_2_ episode was tested using a one-way ANOVA for repeated measurements. Peritumoral and intratumoral regions were compared to the contralateral values using the Scheffé *post hoc* test. Statistical significance threshold was *P*=0.05.

## RESULTS

Of the 26 rats, 17 were excluded from the MRI study for the following reasons: MABP <100 mmHg, Hb <10 g dl^−1^ or pHa <7.30 during control episode (*n*=5); MABP <70 mmHg, PaCO_2_ <27 mmHg or pHa <7.30 during hypoxia episodes (*n*=9), absence of brain tumour (*n*=3). Therefore, the cerebrovascular response to graded hypoxic hypoxia was investigated in nine rats with brain glioma.

Physiological data are shown in Table 1

**Table 1:** Physiological parameters of tumour-bearing rats (*n*=9), at each FiO_2_ level

	**Control**	**Normoxia**	**0.15**	**0.12**	**0.10**	**Reoxygenation**
MABP (mmHg)	115±20	120±15	95±15	90±20	65±20^b^	125±25
PaO_2_ (mmHg)	144.6±16.2	111.2±14.0^b^	56.5±5.8^b^	41.8±4.1^b^	35.4±2.8^b^	154.1±19.0
SaO_2_ (%)	99.2±1.5	94.4±5.7	74.1±5.6^b^	55.2±5.4^b^	43.1±3.9^b^	98.9±2.4
PaCO_2_ (mmHg)	36.0±2.6	35.9±4.8	32.6±2.2	31.1±2.5	28.1±3.3^b^	34.0±2.6
Pha	7.34±0.03	7.35±0.03	7.39±0.02	7.39±0.02	7.37±0.07	7.34±0.07
Hb (g/dl)	13.6±0.9	13.3±1.3	13.3±0.2	13.1±0.7	12.5±0.9	12.9±0.7

a Evolution of mean arterial blood pressure (MABP), arterial blood gases (PaO_2_ and PaCO_2_), arterial saturation of haemoglobin in oxygen (SaO_2_), arterial pH (pHa) and haemoglobin content (Hb) at each episode of FiO_2_ (mean±s.d.).

b *P*<0.05 compared to control value (Scheffé *F*-test).

. Hypoxic hypoxia caused a significant decrease in MABP and hypocapnia at FiO_2_ of 0.10. All parameters returned to control value during reoxygenation episode. Changes in pHa or in Hb were not observed throughout the study.

Typical Δ*R*_2_^*^ images are shown in Figure 3A. Administration of the contrast agent resulted in decrease of the *T*_2_^*^ values, leading to positive Δ*R*_2_^*^ in the brain. The largest Δ*R*_2_^*^ values (corresponding to high BV) were observed in the peritumoral region. The lowest Δ*R*_2_^*^ values were observed in the intratumoral region. Excluded pixels (BV>13.3 ml.100g^−1^) were mainly observed in the peritumoral region at FiO_2_ of 0.35 (Figure 3B). As FiO_2_ was decreased, the number of pixels excluded increased, reaching a maximum at FiO_2_ of 0.10.

**Figure 4:**
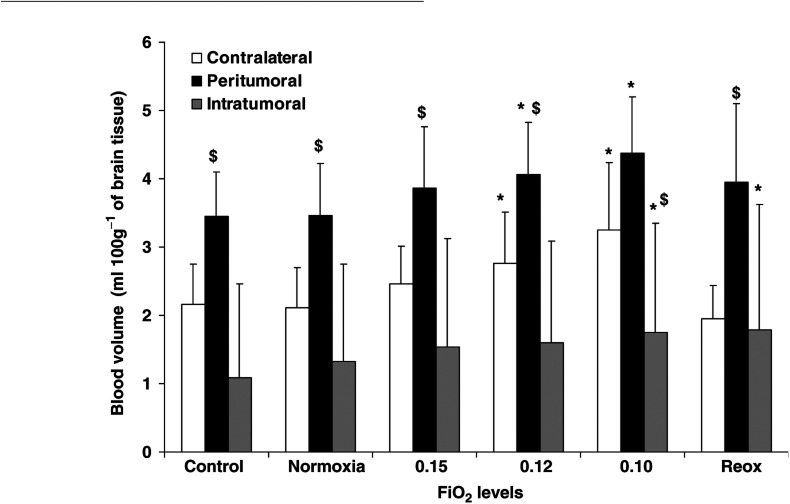
Evolution of blood volume (BV, in ml 100 g^−1^ of brain tissue) in peritumoral, intratumoral and contralateral regions during graded hypoxic hypoxia and reoxygenation (*n*=9). Peritumoral and contralateral BV were reversibly increased during moderate hypoxia (FiO_2_ 0.12) while intratumoral BV was irreversibly increased during severe hypoxia (FiO_2_ 0.10). ^*^P<0.05 between FiO_2_ compared to control value (Scheffé *F*-test). Peritumoral region had a higher BV than contralateral and intratumoral regions. $ *P*<0.05 between ROI compared to contralateral region at each FiO_2_ (Scheffé *F*-test).

There was a significant interaction between brain regions and FiO_2_ episodes (*F*=5.19; *P*<0.01), meaning that a regional difference was found in the response to hypoxia. Interfactor analysis showed that peritumoral BV (3.4±0.7 ml.100 g^−1^) was significantly higher than contralateral (2.2±0.6 ml.100 g^−1^) and intratumoral (1.1±1.4 ml.100 g^−1^) BV during the control episode. In addition, the peritumoral region had a significantly higher BV than the contralateral region for each FiO_2_ episode, apart from FiO_2_ of 0.10 (Figure 4

). The peritumoral and contralateral regions exhibited a significant graded increase in BV during hypoxia, with the greatest degree of hypoxia (FiO_2_ of 0.10) yielding to the largest BV increase. Contralateral BV increases were 28±14% of control value at FiO_2_ of 0.12, and 49±22% at FiO_2_ of 0.10. Peritumoral BV increases were 19±12% of the control value at FiO_2_ of 0.12, and 28±17% at the FiO_2_ episode of 0.10. These changes were reversed during reoxygenation, and the BV returned to control values in the contralateral and the peritumoral regions. It should be noted that no difference in BV changes was found between these two regions during the successive FiO_2_ episodes, including reoxygenation.

A large intervariability in absolute BV values and in BV changes was noted in the intratumoral region (Figure 4). Intratumoral BV significantly increased only at a FiO_2_ of 0.10 (+186±253% of control values), although the absolute BV values were significantly lower than the contralateral BV at this level of hypoxia. Moreover, no evidence of return toward control values was found in this region during the reoxygenation.

**Figure 5:**
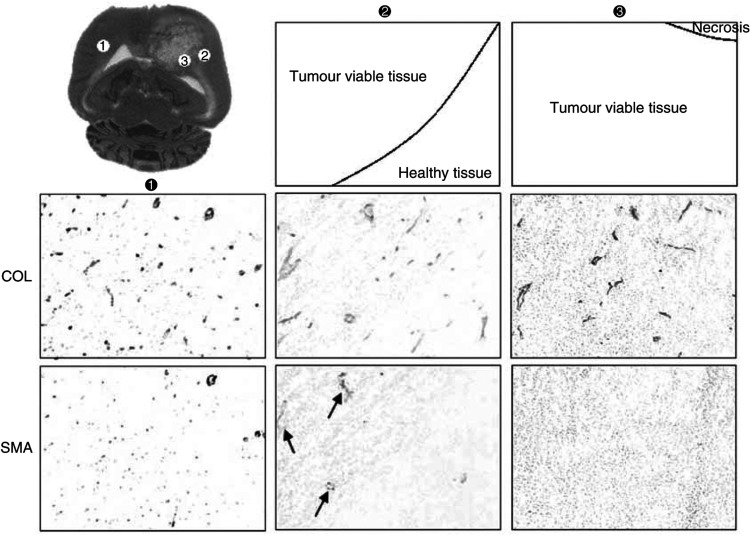
Immunohistochemistry detection in rat brain tumour in 
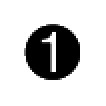
 contralateral, 
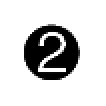
 peritumoral (at the border between normal and viable tumour tissues) and 
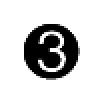
 intratumoral (viable tumour tissue near necrosis) regions. Histological staining was performed by HES. All vessels were stained using anti-collagen antibody (COL) and mature vessels were stained using anti-*α* smooth muscle actin antibody (SMA). Some vessels in the peritumoral region exhibited both COL and SMA staining (indicated by arrows) suggesting reactive vessels to hypoxic hypoxia.

Histological analysis confirmed that the C6 glioma developed at the expense of striatal tissue. The size of the brain tumour was not different between the rats. The peritumoral region in the MRI experiment corresponded to the limit between normal tissue and viable, non-necrotic, intratumoral tissue. Collagen and *α*-SMA staining were used to qualitatively assess the number and morphology of all vessels and of the smooth muscle cells and pericytes characterising mature vessels respectively (Figure 5

). In the peritumoral tissue, there were numerous vessels, mostly stained using *α*-SMA. In the intratumoral region, few, large and tortuous vessels with no *α*-SMA staining were depicted in the viable tumour tissue. There were no vessels in the necrotic areas.

## DISCUSSION

Using the *in vivo* measurement of regional BV in a model of rat brain glioma, we found a comparable vascular response to graded hypoxic hypoxia of the peritumoral and contralateral regions. Combined with the histological study showing *α*-SMA staining in the peritumoral region, these findings indicate that vessels in this region present intact vasoreactivity. Similar results using MRI and SMA staining were reported in a model of subcutaneous tumour exposed to hypercapnia (Abramovitch *et al*, 1999). This vascular reactivity could be taken advantage of to improve the delivery of antitumoral agent through the increase of local blood flow.

Some experimental points need to be addressed. First, despite epinephrine, the highest level of hypoxic hypoxia (FiO_2_ of 0.10) was associated with hypocapnia and with a decrease in MABP, which *a priori*, might interfere with BV changes. However, as discussed elsewhere (Julien-Dolbec *et al*, 2002), the influence of these two parameters on BV changes is likely minor compared to that of severe hypoxia hypoxia, and should not invalid the comparison between regions of the response to hypoxia. Second, the relationship between Δ*R*_2_^*^ and BV postulates that the contrast agent must be confined within the vascular space. This particular point has been addressed by Dennie *et al* (1998), who showed that Δ*R*_2_^*^ did not change in rat brain tumour during 90 min following the administration of a contrast agent (MION) very similar to the present one (AMI-227/Sienerem®). Since our experiment lasted about 75 min, we considered that the contrast agent extravasation was negligible, regardless the status of the blood–brain barrier. The restoration of BV measurements to control values during the reoxygenation period suggests that extravasation does not occur in peritumoral region. Conversely, a leakage of contrast agent in tumoral region cannot be definitively ruled out.

The difference in the control values between the three brain regions can be due to a difference in their vessel density. Indeed, the large BV value found in the peritumoral region agrees with the high density of vessels reported by others (Deane and Lantos, 1981; Luthert and Lantos, 1985; Dennie *et al*, 1998; Peoc'h *et al*, 1999). Conversely, the low BV values found in the intratumoral region are consistent with the presence both of viable tumour tissue with few vessels and necrosis without vessels. A quantitative study was previously conducted to explore vessel density in the same model of rat brain tumour (Peoc'h *et al*, 1999), and our results are in agreement with this quantitative study. However, our control values of BV in the contralateral region (2.2±0.6 ml.100 g^−1^) were notably smaller than those found in normal rat striatum using the same technique (3.1±0.7 ml.100 g^−1^) (Julien-Dolbec *et al*, 2002). Similar findings concerning local cerebral blood flow were noted between normal rats and rats with brain glioma (Aizawa *et al*, 1990). These differences could be due to a tumour-related compression of surrounding tissues, particularly during the late stage of tumour growth.

It is established that hypoxic hypoxia is accompanied in the healthy brain by an increase in cerebral blood flow (Koehler *et al*, 1984). This increase is due to dilation of the cerebral arterioles (Kontos *et al*, 1978). Considering that pixels with high BV (i.e., BV>13.3 ml.100g-1) were excluded (see Materials and Methods section), the present changes in BV in the contralateral tissue should involve parenchymal arterioles and venules. The BV response to hypoxic hypoxia in this region (+28±14% of control value at FiO_2_ of 0.12) was close to that reported in normal rats (+38±25%) (Julien-Dolbec *et al*., 2002).

In brain tumour, there is a large heterogeneity in vessel morphology (number, size, connections). Previous studies (Abramovitch *et al*, 1999; Holash *et al*, 1999) indicated however that vessels located between normal and tumoral tissue have mostly *α*-smooth muscle actin staining (as also shown in this study) and thus correspond to mature pre-existing vessels. Our results, showing comparable hypoxia-induced changes in BV in the peritumoral and the contralateral regions, are in line with these *in vitro* findings. Conversely, intratumoral tissue contains immature vessels and/or necrosis. The large, tortuous vessels with no *α*-smooth muscle actin likely correspond to angiogenesis-induced neovessels (Deane and Lantos, 1981; Zama *et al*, 1991). In the present study, a significant change in intratumoral BV was found during severe hypoxic hypoxia (FiO_2_ of 0.10) only, with no return towards control values during reoxygenation. This can be explained by a possible extravasation of the contrast agent and/or a passive, hypoxic-induced vasodilation resulting from surrounding vasodilated tissue (Robinson *et al*, 1999). The large variability in BV values and BV changes in this region may be ascribed to a redistribution of the perfusion within the tumour (Dewhirst *et al*, 1989; Kuperman *et al*, 1995).

In conclusion, our study shows that the vascular response to a vasodilating stimulus such as hypoxic hypoxia can be measured *in vivo* in a model of rat brain glioma. Peritumoral vessels had a response to hypoxic hypoxia that was comparable to that from normal vessels. Susceptibility contrast MRI is an appropriate method for noninvasively demonstrating whether any particular vascular-modifying strategy results in the desired outcome in terms of tumour blood volume changes.
